# Enhanced Gastrointestinal Expression of Cytosolic Malic Enzyme (ME1) Induces Intestinal and Liver Lipogenic Gene Expression and Intestinal Cell Proliferation in Mice

**DOI:** 10.1371/journal.pone.0113058

**Published:** 2014-11-17

**Authors:** Ahmed Al-Dwairi, Adam R. Brown, John Mark P. Pabona, Trang H. Van, Hamdan Hamdan, Charles P. Mercado, Charles M. Quick, Patricia A. Wight, Rosalia C. M. Simmen, Frank A. Simmen

**Affiliations:** 1 Department of Physiology & Biophysics, University of Arkansas for Medical Sciences, Little Rock, AR, United States of America; 2 Department of Pathology, University of Arkansas for Medical Sciences, Little Rock, AR, United States of America; 3 Interdisciplinary Biomedical Sciences Program, University of Arkansas for Medical Sciences, Little Rock, AR, United States of America; 4 The Winthrop P. Rockefeller Cancer Institute, University of Arkansas for Medical Sciences, Little Rock, AR, United States of America; IRCCS Istituto Oncologico Giovanni Paolo II, Italy

## Abstract

The small intestine participates in lipid digestion, metabolism and transport. Cytosolic malic enzyme 1 (ME1) is an enzyme that generates NADPH used in fatty acid and cholesterol biosynthesis. Previous work has correlated liver and adipose ME1 expression with susceptibility to obesity and diabetes; however, the contributions of intestine-expressed ME1 to these conditions are unknown. We generated transgenic (Tg) mice expressing rat ME1 in the gastrointestinal epithelium under the control of the murine villin1 promoter/enhancer. Levels of intestinal ME1 protein (endogenous plus transgene) were greater in Tg than wildtype (WT) littermates. Effects of elevated intestinal ME1 on body weight, circulating insulin, select adipocytokines, blood glucose, and metabolism-related genes were examined. Male Tg mice fed a high-fat (HF) diet gained significantly more body weight than WT male littermates and had heavier livers. ME1-Tg mice had deeper intestinal and colon crypts, a greater intestinal 5-bromodeoxyuridine labeling index, and increased expression of intestinal lipogenic (*Fasn, Srebf1*) and cholesterol biosynthetic (*Hmgcsr*, *Hmgcs1*), genes. The livers from HF diet-fed Tg mice also exhibited an induction of cholesterol and lipogenic pathway genes and altered measures (*Irs1*, *Irs2, Prkce*) of insulin sensitivity. Results indicate that gastrointestinal ME1 via its influence on intestinal epithelial proliferation, and lipogenic and cholesterologenic genes may concomitantly impact signaling in liver to modify this tissue’s metabolic state. Our work highlights a new mouse model to address the role of intestine-expressed ME1 in whole body metabolism, hepatomegaly, and crypt cell proliferation. Intestinal ME1 may thus constitute a therapeutic target to reduce obesity-associated pathologies.

## Introduction

The prevalence of obesity and its associated comorbidities has greatly increased over the past several decades [1]. The small intestine is an important organ for digestion, absorption and transport of dietary lipids, and thus, may play an important role in conferring obesity and related pathologies [2], [3]. Previous studies have shown that expression of cytosolic malic enzyme (ME1) and other lipogenic genes is elevated in the small intestine in animal models of diet-induced obesity [2], [4], [5] and that fatty acid and cholesterol biosynthesis and transport are up-regulated in the small intestine of diabetic, obese rats [6], [7]. Moreover, germ-free mice are protected from diet-induced obesity, suggesting the influence of host-microbe interactions on intestinal lipid metabolism [8]. The collective findings point to an important role for intestinal metabolic pathways in the regulation of body glucose and lipid metabolism, and in the etiology of obesity and diabetes. Nevertheless, a direct role for a specific intestinal lipid metabolic gene in the development of these conditions has not been established.

In addition to participating in fatty acid synthesis, lipogenic enzymes are important for the proliferation of epithelial cells by supplying cell membrane components, and in the maintenance of the intestinal epithelial barrier [9]. These latter functions may affect the overall absorptive surface area available as an adaptation to diet, and when deregulated may potentially promote intestinal epithelial neoplastic transformation [10].

ME1 is a major lipogenic enzyme that catalyzes the oxidative decarboxylation of malate to pyruvate and concomitantly reduces NADP^+^ to NADPH. The latter contributes to a pool of NADPH that is used in fatty acid and cholesterol biosynthetic (as well as other) pathways, and specifically in the rate-determining steps catalyzed by Fatty Acid Synthase (FASN) and HMG-CoA Reductase (HMGCR), respectively [11], [12]. However, ME1 is not the sole source of reduced NADPH in this regard. ME1 is ubiquitously expressed [13] although at variable levels in different tissues, is moderately expressed in gastrointestinal epithelium and smooth muscle, and in liver is subject to transcriptional regulation by insulin and thyroid hormone receptor pathways [14]. Mice, unlike rats and humans, express two ME1 protein variants due to differential RNA splicing [13].

Previous studies have reported robust associations of liver and adipose tissue ME1 with susceptibility to both diabetes and obesity in humans and rodent models [15]–[17]. In this regard, mice that are functionally null for ME1 (MOD-1 mouse line) are protected from diet-induced obesity and hepatosteatosis, and had reduced serum insulin and leptin concentrations as well as lowered levels of proliferation markers in the small intestine [15], [16]. However, the direct contribution of intestinal ME1 to these conditions is unknown.

Here, we generated transgenic mice with enhanced expression of ME1 in the intestinal epithelium via use of the mouse villin1 gene promoter-enhancer, in order to investigate the effects of augmented intestinal ME1 expression on epithelial proliferation and tissue morphology, gastrointestinal lipogenic and cholesterogenic genes, and the development of obesity and hepatosteatosis. We hypothesized that an increased level of intestinal ME1 would promote intestinal cell proliferation and lipogenic pathway gene expression, as well as induce metabolic alterations and predisposition to obesity and hepatosteatosis. We now report that Tg mice expressing rat ME1 in the intestinal epithelium when fed high-fat (HF) diet, manifest larger livers as well as up-regulation of fatty acid and cholesterol biosynthetic pathway genes in both small intestine and liver.

## Materials and Methods

### Generation of Tg mice expressing rat ME1 in the small intestine epithelium

**Figure 1:**
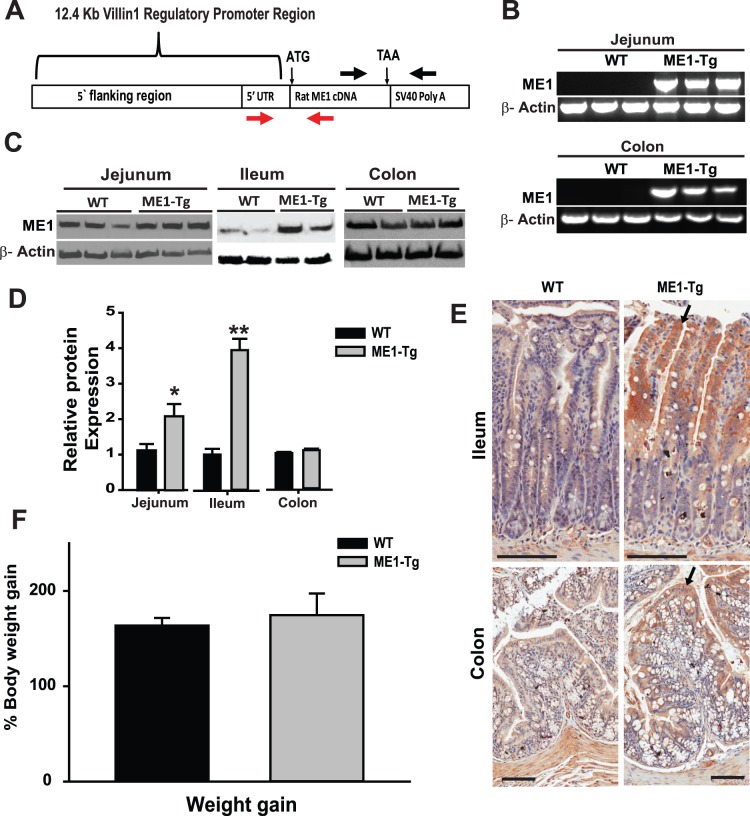
ME1-Tg mice on chow diet exhibit increased ME1 protein and mRNA abundance in small intestine. A) Schematic representation of the mouse villin1-ME1 transgene construct in which a complete open reading frame for rat ME1 was placed downstream of the murine villin1 gene promoter-enhancer (12.4 kb fragment). The SV40 polyA signal-containing region was located downstream of the *Me1* cDNA sequence. Red arrows indicate the location of genotyping primers, while black arrows indicate the location of primers used to detect transgene expression by RT-PCR. B) ME1-Tg mRNA expression in the jejunum and colon of WT and ME1-Tg mice detected by RT-PCR (n = 3 mice/group; Exp.1). C) Representative Western blots of ME1 protein in the jejunum, ileum and colon of WT and ME1-Tg mice (n = 2−3/group; Exp. 1). The ME1 antibody detected endogenous murine and Tg-derived rat ME1 proteins. D) Densitometric analysis of relative ME1 protein levels in panel C. E) Representative images of immunohistochemical staining of ME1 in the Ileum and colon of WT and ME1-Tg Tg mice. Scale bars = 100 µM. Arrows indicate villous epithelial and luminal epithelial staining of ME1 in the ileum and colon, respectively. F) Weight gain calculated as percentage increase of final body weight from initial body weight of WT and ME1-Tg male mice (n = 8−10 mice/group) from Exp. 1. Bar graphs represent mean ± SEM; *Significant difference at *P*<0.05 between genotypes. **Significant difference at *P*<0.01 between genotypes. *P* values are indicated for those tending to have significant differences between groups (0.05<*P*<0.10).

All animal procedures were approved by the University of Arkansas for Medical Sciences Animal Care and Use Committee. Intestine-specific expression of rat ME1 was driven by the villin1 promoter-enhancer as illustrated in Figure 1A. The vil-Me1 mouse transgene consisting of a 12.4 kb villin1 promoter-enhancer fragment fused to full length rat *Me1* cDNA, was generated as follows. The malic enzyme 1 cDNA (1.8 kb) was excised from a Topo cloning Vector (Invitrogen, Carlsbad, CA) via double digestion with *Kpn I* and *Xho I*, resolved in an agarose gel, and purified using a gel fragment purification kit (Qiagen, Valencia, CA). A villin1 promoter-enhancer construct (from D. Gumucio, Univ. Michigan) was sequentially digested with *Kpn I* and *Xho I* and the desired fragment purified from a gel slice via electroelution. Ligation reactions of villin1 promoter-enhancer DNA and *Me1* cDNA utilized a 1∶3 vil:Me1 ratio and T4 ligase (New England Biolabs, Ipswich, MA) at 14°C for 16 hours. Transformation of electrocompetent *E. coli* cells (Invitrogen) was carried out. Colonies were picked, grown in 2 mL Luria-Bertani Broth, DNA isolated using a miniprep kit (Zymo, Irvine, CA), and DNA was digested with *EcoRI* and *XhoI/KpnI* enzymes for diagnostic purposes. The final vil-Me1 plasmid was digested with *PmeI* (New England Biolabs) to remove a 2.5 kb segment containing vector sequence and the remaining 17.3 kb DNA fragment was purified. The latter was microinjected into the pronuclei of fertilized eggs of C57BL/6J mice. Transgenic founders were identified by PCR of genomic DNA obtained from tail biopsies. Primers amplified a 330 bp fragment spanning the villin1 promoter and Me1 coding region (forward primer: 5′-AAG GAT CAT CAT CAA AGC CGG GTG-3′, and reverse primer: 5′-GCC ATG AAT GTT CAG CTG TTG CCT-3′).

### Animals, diets, and tissue collection

Animals were maintained on a 12 h light-12 h dark cycle, and were assigned to one of two diets. In Experiment 1 (Exp. 1; initial characterization of transgenic line), male WT and ME1-Tg mice were fed a standard mouse chow diet (Harlan Laboratories, Madison, WI) after weaning (3 wk of age, n = 8 per group), and were monitored for body weight for 8 wk (final age = 11 wk), at which time they were euthanized. In Exp. 2, weanling male WT and ME1-Tg mice were fed a HF diet (45% kcal from fat, Harlan; Table 1) for 15 wk to maximize the obesogenic effect of diet (final age = 18 wk, n = 10 per group), at which time they were euthanized. Mice were provided food and water *ad libitum* and were monitored weekly for body weight. Animals were euthanized between 8–11 a.m., 3 h after food withdrawal. Sera and tissues [small and large intestines, frontal lobe of the liver, gonadal (GF) and retroperitoneal fat (RPF) pads] were collected. The small intestine was divided into 3 equal parts; these were designated duodenum, jejunum, and ileum. The junction between jejunum and ileum (∼1 cm) was fixed in 10% formalin and embedded in paraffin for later histological analysis. The large intestine was divided into 3 equal parts: proximal, distal (used for RNA and protein analysis), and middle section (fixed in formalin). Tissues were snap frozen in liquid nitrogen and stored at −80°C until use. Mice in Exp. 2 were injected intra-peritoneal with 5-bromodeoxyuridine (BrdU) (Sigma Aldrich, St. Louis, MO) at a dose of 100 mg/kg (body weight; BW) at 2 h prior to euthanasia, in order to evaluate gastrointestinal tract proliferative status. Jejunums were obtained from MOD-1 and WT mouse counterparts fed HF diet as described previously [15].

**Table 1:** Composition of experimental HF diet.

Formula	g/Kg
Casein	210.0
L-Cystine	3.0
Corn Starch	130.0
Maltodextrin	180.0
Sucrose	150.0
Lard	210.0
Corn Oil	20.0
Cellulose	28.96
Mineral Mix, AIN-93G-MX (94046)	50.0
Vitamin Mix, AIN-93-VX (94047)	15.0
Choline Bitartrate	3.0
TBHQ, antioxidant	0.04

Approximately 45% of total calories derived from fat, with casein as the protein source. Kcal/g = 4.7.

### RNA isolation and quantitative real time/reverse transcriptase-polymerase chain reaction (qRT-PCR)

RNA was extracted from individual jejunum, ileum, distal colon and liver samples (n = 7−9 mice/group/tissue) with TRIzol (Life Technologies, Grand Island, NY). One microgram of RNA was reverse-transcribed to obtain cDNA using the iScript cDNA synthesis kit (Bio-Rad; Hercules, CA). Expression of target genes was assayed by qRT-PCR using Bio-Rad iTaq SYBR Green Supermix. PCR primers (Table 2) were obtained from Integrated DNA Technologies, Inc. (Coralville, IA). Target mRNA abundance was normalized to a factor derived from the geometric mean of expression values for mouse β-Actin (*Actb*), Cyclophilin A (*Ppia*) and TATA box binding protein (*Tbp*), calculated using GeNorm [18]. Student’s *t*-test was used to compare variables between groups (SigmaPlot 12; Systat Software, Inc.; Chicago, IL). To detect expression of the ME1 transgene in the colon and small intestine by conventional RT-PCR, a primer pair was used to amplify a segment spanning the SV40 poly A and ME1 coding regions [forward primer (Me1): 5′-AAT GAT TCG GTC TTC CTC ACC-3′, and reverse primer (SV40): 5′-CAG ACA TGA TAA GAT ACA TTG ATG AGT T-3′] of the transgene construct.

**Table 2:** RT-PCR primers.

mus *Angptl4*	NM_020581.2	GACTTTTCCAGATCCAGCCTC	CTCCGAAGCCATCCTTGTAG
mus *ApoA1*	NM_009692.4	TGTGTCCCAGTTTGAATCCTC	GTTATCCCAGAAGTCCCGAG
mus *ApoE*	NM_009696	CAATTGCGAAGATGAAGGCTC	TAATCCCAGAAGCGGTTCAG
mus *Actb*	NM_007393.3	ACCTTCTACAATGAGCTGCG	CTGGATGGCTACGTACATGG
mus *Cyp4a10*	NM_010011.3	CCCAAGTGCCTTTCCTAGATG	GCAAACCATACCCAATCCAAG
mus *Fasn*	NM_007988.3	CCCCTCTGTTAATTGGCTCC	TTGTGGAAGTGCAGGTTAGG
mus *v-Fgr*	NM_010208.4	CATAGAGGACAATGAGTATAACCCC	AGTGAGCAGAATCCCAAAGG
mus *Hmgcr*	NM_008255.2	AGTCAGTGGGAACTATTGCAC	TTACGTCAACCATAGCTTCCG
mus *Hmgcs1*	NM_145942.4	TGTTCTCTTACGGTTCTGGC	AAGTTCTCGAGTCAAGCCTTG
mus *Irs1*	NM_010570	ATAGCGTAACTGGACATCACAG	GCATCGTACCATCTACTGAAGAG
mus *Irs2*	NM_001081212	GTCCAGGCACTGGAGCTTT	GCTGGTAGCGCTTCACTCTT
mus *Ldlr*	NM_010700.3	ACCCGCCAAGATCAAGAAAG	GCTGGAGATAGAGTGGAGTTTG
mus *Lpl*	NM_008509.2	AACAAGGTCAGAGCCAAGAG	CCATCCTCAGTCCCAGAAAAG
mus *Lepr*	NM_146146.2	ATTTCCTCTTGTGTCCTACTGC	AAGATGCTCAAATGTTTCAGGC
mus *Me1*	NM_008615.2	AGTATCCATGACAAAGGGCAC	ATCCCATTACAGCCAAGGTC
mus *Pparγ*	NM_011146	TGTTATGGGTGAAACTCTGGG	AGAGCTGATTCCGAAGTTGG
mus *Ppia*	NM_008907.1	GCAGACAAAGTTCCAAAGACAG	CATTATGGCGTGTAAAGTCACC
mus *Prcke*	NM_011104	GCCATCAAGCAACATCCATTC	TGTAAGTATTGGCTCTTCCCG
mus *Rxrγ*	NM_009107	CAGAGTCCTTACAGAGTTGGTG	TCGAAGAGTCTCCACCTCAG
mus *Scd1*	NM_009127	CTGACCTGAAAGCCGAGAAG	AGAAGGTGCTAACGAACAGG
mus *Srebf1*	NM_011480.3	CCATCGACTACATCCGCTTC	GCCCTCCATAGACACATCTG
mus *Tbp*	NM_013684.3	AAGAAAGGGAGAATCATGGACC	GAGTAAGTCCTGTGCCGTAAG
rat *Actb*	NM_031144.3	CAGCCTTCCTTCCTGGGTATG	TAGAGCCACCAATCCACACAG
rat *Ppia*	NM_017101.1	CCCACCGTGTTCTTCGACAT	TCTCCCAGTGTCAGAGCA
rat *Fasn*	NM_017332.1	AATTGCCCGAGTCAGAGAAC	ACAGATCCTTCAGCTTTCCAG
rat *Lpl*	NM_012598	CTTAGGGTACAGTCTTGGAGC	CATCAGGAGAAAGGCGACTAG
rat *Rxrγ*	NM_031765.1	GGGAGCGAGCAGAGAGTGAGG	GTGGGGGATGCGTTTGGC
rat *Scd1*	NM_139192	TCTTCATCGACTGCATGGC	TGGAACAGGAACTCAGAAGC
human *ME1*	NM_002395.5	ATCCTCAAGAATGTCTGCCTG	GCCGTAGTCCAATGTAGAGTG

### Protein isolation and Western blot analysis

Jejunums, ileums, and livers were homogenized in RIPA buffer containing protease and phosphatase inhibitors (Santa Cruz Biotechnology; Santa Cruz, CA) and protein concentrations were determined using the bicinchoninic acid (BCA) protein assay kit (Pierce; Rockford, IL). Proteins were separated in 10% (for ME1 and LPL, 40 µg) or 8% (for FASN, 40 µg; IRS1/IRS2, 15 µg) sodium dodecyl sulfate-polyacrylamide gel electrophoresis (SDS-PAGE), and transferred to a nitrocellulose membrane. Membranes were incubated overnight with rabbit polyclonal antibody raised against ME1 (ab97445, 1∶1000 dilution; Abcam, Cambridge, MA), rabbit polyclonal antibody raised against FASN (ab22759, 1∶1000 dilution; Abcam, Cambridge, MA), rabbit polyclonal antibody raised against IRS1 (PA1-1057, 1∶500 dilution; Thermo Scientific), rabbit polyclonal antibody raised against pSer307-IRS1 (PA1-1054, 1∶500 dilution; Thermo Scientific), rabbit polyclonal antibody raised against IRS2 (4502P, 1∶1000 dilution, Cell Signaling), mouse monoclonal antibody raised against LPL (ab21356, 1∶1000 dilution; Abcam), rabbit polyclonal antibody raised against α-Tubulin (sc-5546, 1∶1000 dilution, Santa Cruz Biotechnology) or mouse monoclonal antibody raised against β-actin (A1978, 1∶10,000 dilution; Sigma Aldrich). Antibodies were incubated with membranes in Odyssey blocking buffer (Li-Cor Biosciences; Lincoln, NE). Membranes were then placed in blocking buffer for 1 h, washed and incubated with appropriate horseradish peroxidase-conjugated secondary antibodies for 1 h. Proteins were visualized using chemiluminescence (Amersham Bioscience; Piscataway, NJ). Images were acquired with a FlourChem HD2 Imager system (Alpha Innotech, San Leandro, CA) and densitometry was performed using Alpha View software (Cell Biosciences; Santa Clara, CA). Levels of ME1, IRS1, and IRS2 were normalized to β-actin while FASN was normalized to α-tubulin. Level of Ser307 phosphorylation was calculated by dividing band density of pSer307-IRS1 by that of total IRS1.

### Serum hormone and glucose concentrations

Blood was centrifuged to obtain serum. Blood glucose levels were measured from tail blood while the animals (12 wk of age) were under isoflurane anesthesia and from trunk blood after euthanasia (18 wk of age), using a Glucometer (One Touch Ultra Blood Glucose Monitoring System, Lifescan, Milpitas, CA). Serum insulin, leptin, and adiponectin (n = 6–8 animals/group) were quantified using Milliplex MAP Mouse Adipokine kits (Millipore Corp., Billerica, MA). Each individual mouse serum sample was assayed in duplicate. Homeostatic Model Assessment – Insulin Resistance (HOMA-IR) was calculated using the equation (fasting blood glucose × fasting blood insulin/22.5), as previously described [19].

### Histology and immunohistochemistry

Jejunum (jejunum-ileum junction) and mid-colon were fixed in 10% neutral-buffered formalin (pH 7.4) overnight and were then embedded in paraffin. Five µm sections were stained with hematoxylin/eosin (H&E). For analyses by immunohistochemistry (IHC), paraffin-embedded samples were sectioned, and the sections were dewaxed and rehydrated through a graded alcohol series as previously described [20]. Antigen unmasking was performed by boiling the sections in Citra Plus (Biogenex, San Ramon, CA) in a microwave oven for 2 min at power 10 and then for 10 min at power 1, followed by cooling for 20 min. Sections were treated with 3% hydrogen peroxide to quench endogenous peroxidase activity and incubated in blocking solution containing goat IgG (Vectastain Elite ABC kit, Vector Laboratories, Inc.; Burlingame, CA, USA) for 30 min. Sections were incubated overnight with rabbit ME1 polyclonal antibody (16619-1-AP, 1∶200 dilution, Proteintech Group, Inc.; Chicago, Illinois), mouse monoclonal antibody raised against LPL (ab21356, 1∶100 dilution; Abcam), or rat monoclonal anti-BrdU antibody (ab6326, 1∶40 dilution Abcam) followed by incubation with goat anti-rabbit secondary antibody (Vectastain Elite ABC kit; Vector Laboratories) for 30 min. Sections were stained with 3,3′-diaminobenzidine tetra-hydrochloride (Dako Inc.; Carpinteria, CA) and counterstained with hematoxylin; slides were then dehydrated in an alcohol series and cleared in xylene. Images were acquired and analyzed with Aperio ImageScope (Aperio Technologies Inc.; Vista, CA). Jejunum crypt depth and villus height were measured from 6–10 crypt-villus units per animal (5–6 individual mice/group). BrdU-labeled cells were counted in 10–15 crypts per mouse (5 mice/group).

### Oil Red O staining and Cholesterol Assay

Liver samples to be used for analysis of lipid content were frozen in OCT compound (Sakura Finetek USA, Inc., Torrance, CA), sectioned at 7 µm thickness, stained with Oil Red O, and counterstained with hematoxylin. Lipid droplet staining intensity in sections from 9 mice/group was determined using Aperio ImageScope software. Formalin-fixed, paraffin-embedded liver tissue from the same animals was sectioned and H&E-stained.

Serum and liver homogenate cholesterol levels were assayed using a Cholesterol Fluorometric Assay Kit according to the manufacturer’s protocol (Cayman Chemical #10007640; Ann Arbor, Michigan).

### ME1 activity assay

ME1 enzyme assay was performed following published methods [21], [22]. In brief, jejunum was homogenized in 20 mM HEPES, pH 7.5, 10 mM KCl, 1.5 mM MgCl_2,_ 1 mM sodium EDTA, 1 mM dithiothreitol, 250 mM sucrose and protease inhibitors. Homogenates were centrifuged at 14,000 rpm for 20 min at 4°C, and supernatant was collected. ME1 activity was determined by monitoring the formation of NADPH in a reaction buffer containing 67 mM triethanolamine, 3.3 mM L-malate, 0.3 mM β-NADP^+^ and 5 mM MgCl_2_ in 96-well plate format. Results are presented as nmol NADPH/µg protein.

### Tissue NADPH/NADPt measurements

Tissue NADPH and NADP^+^ levels were measured using a NADP^+^/NADPH quantification kit (BioVision). Jejunum was homogenized in NADPH extraction buffer (kit) and a portion of the homogenate was heated to 60°C for 30 min; conditions that decompose NADP^+^ but not NADPH. Homogenates were mixed with cycling enzyme mix, and a colorimetric reaction was used to measure absorbance of both NADP^+^ and NADPH for each heated and unheated fraction at a wavelength of 450 nm. Data are presented as the ratio of NADPH to total (NADP^+^+NADPH) levels (pmol) per milligram of protein.

### Cell culture, transfection and proliferation assay

The rat intestinal epithelial cell line IEC-6 (CRL-1592, obtained from American Tissue Culture Collection; Manassas, VA) was propagated in DMEM medium supplemented with 10% heat-inactivated fetal bovine serum (FBS; Hyclone, Fisher) and bovine insulin (Sigma) in a 5% CO_2_ incubator at 37°C. For transfection, cells (2×10^4^/well) plated in 24-well plates in complete media, were transfected 24 h later with an expression vector for human ME1 (pCMV6-XL4-hME1) or with a control expression vector (pCMV6-XL4) (Origene; Rockville, MD), using Effectene transfection reagent (Qiagen). Cells were allowed to grow for 48 h, and proliferation was evaluated by MTT assay (Promega; Madison, WI). In a separate experiment, 2×10^5^ cells per well were plated in 6-well culture plates in complete medium for 24 h, and then transfected with ME1 expression or control vectors (as above). After 96 h, cells were lysed in TRIzol reagent, and RNA was collected for qRT-PCR analysis. Transcripts were normalized to rat β-Actin (*Actb*) and Cyclophilin A (*Ppia*) (primer sequences shown in Table 2).

### Statistics

Values shown represent the means ± SEM. Statistical analysis between groups was by Student’s *t*-test (SigmaPlot; Systat Software, Inc.; Chicago, IL). *P*<0.05 was considered to be statistically significant, while 0.05<*P*<0.1 was considered to represent a tendency for a difference.

## Results

### Generation and characterization of ME1-Tg mice

Intestine-specific expression of rat ME1 was accomplished by use of the murine villin1 promoter-enhancer (Figure 1A). Transgenic founders were confirmed by genotyping (Figure 1A). One transgenic founder was used for establishment of a transgenic line, which was subsequently used for all experiments described here. This line (designated ME1-Tg) was studied in parallel with non-transgenic (WT) littermates and was found to manifest intestinal expression (jejunum, colon) of chimeric mRNA spanning the coding region of ME1 and the SV40 polyA region (Figure 1B).

In Exp. 1, we characterized body weight accretion of ME1-Tg and WT control animals when placed on standard mouse chow. Increased levels of ME1 protein in ME1-Tg relative to WT controls was confirmed by Western blot analysis of different segments of the small intestine (jejunum and ileum) from Exp. 1 animals (Figure 1C). The immunoreactive protein is the sum of endogenous murine and Tg rat ME1 proteins, since the antibody used in Western blot analysis cross reacts with both. Densitometry of immunoreactive bands revealed a significant increase in ME1 protein levels in the jejunum (2-fold, *P*<0.05) and ileum (4-fold, *P*<0.01) of ME1-Tg compared to WT littermates (Figure 1D). However, colon displayed no difference in ME1 levels between ME1-Tg and WT controls by Western blot. Immunohistochemical staining of the ileum for ME1 showed greater staining in villous epithelium, which was primarily localized to the upper villus halves, in Tg *vs.* control mice (Figure 1E). Colon sections routinely displayed more ME1 staining in upper cryptal epithelium and luminal epithelium of ME1-Tg compared to WT mice, in agreement with the previously described pattern of reporter gene expression with the promoter-enhancer region used here [23], [24]. However, the increase in immunoreactivity in colon epithelium was less robust than that found for ileum of Tg *vs.* WT animals (Figure 1E). Animals of both genotypes had comparable amounts of staining for ME1 in intestinal smooth muscle (*muscularis externa*).

To evaluate effects of enhanced ME1 expression on body and tissue weights, WT and ME1-Tg mice were fed standard chow diet for 8 wk after weaning and were monitored for weight gain (Exp. 1). At weaning, WT and ME1-Tg animals were of similar weights. At the end of the study, male Tg mice displayed no significant difference in final body weight increase compared to WT littermates (mean ± SEM = 174.6% ±22.7 *vs.* 163.7% ±7.96, *P* = 0.66) (Figure 1F). No significant changes in liver or fat depot [gonadal fat (GF), retroperitoneal fat (RPF)] weights were noted between the two genotypes (Figure S1A–C). Concentrations of blood glucose, and serum insulin and serum leptin did not also differ between Tg and WT mice at study termination (Figure S1D–F). qRT-PCR analysis showed no significant alterations in the mRNA abundance of *Me1, Fasn, Srebf1, Pparg, Hmgcr*, *Hmgcs1*, *Ldlr, Apoa1, Apoe* and *Cyp4a10 genes*, while *Prkce* transcript levels were decreased in livers of ME1-Tg compared to WT controls (Figure S2A–B).

### Intestinal ME1 overexpression increases body and liver weights during diet-induced obesity

**Figure 2:**
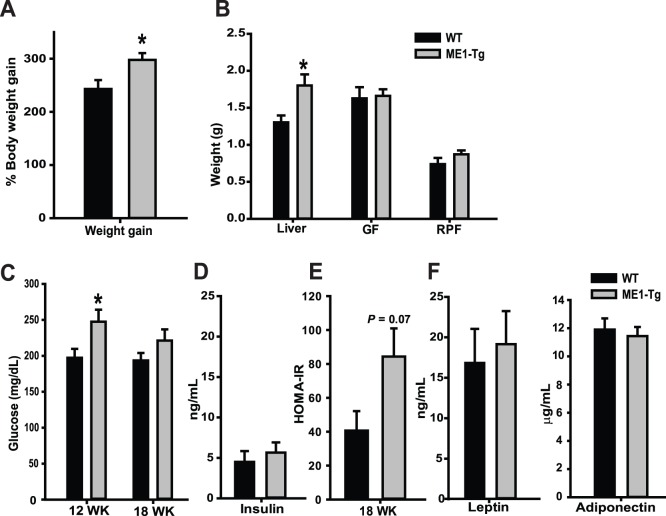
Enhanced intestinal ME1 expression promotes weight gain during consumption of HF-diet. (A) Body and (B) liver, gonadal (GF) and retroperitoneal (RPF) fat depot weights of WT and ME1-Tg mice (n = 10 mice/group) from Exp. 2. Weight gain (A) was calculated as percentage increase of final body weight from initial body weight. C) Fasting (3–4 h) serum glucose levels at 12 wk and 18 wk of age in WT and ME1-Tg mice (Exp. 2). D) Serum levels of insulin, (E) HOMA-IR index mice (at 18 wk), and F) serum leptin and adiponectin levels of WT and ME1-Tg (at 18 wk); n = 7−8 mice/group for C–F. Bar graphs present mean ± SEM; * Significant differences at *P*<0.05 between genotypes. *P* value was indicated for those tending to have significant differences between groups (0.05<*P*<0.10).

To examine effects of enhanced gastrointestinal ME1 expression in the context of excess dietary fat, male WT and ME1-Tg mice were fed a diet high in fat (HF; 45% kcal from fat) beginning at weaning and continuing for a period of 15 wk (postnatal wk 3–18) (Exp. 2). ME1-Tg mice displayed a modest increase in final body weight (36.4±1.5 g for WT *vs.* 41.1 g ±1.9 g for Tg, *P* = 0.07), and a significant, albeit small increase in % weight gain (1.2-fold, *P* = 0.03) compared to WT littermates (Figure 2A). ME1-Tg mice also displayed a significant increase in liver weights (1.3±0.09 g for WT *vs.* 1.8±0.15 g for Tg, *P* = 0.01), without changes in GF or RPF depot weights (Figure 2B).

We next examined for effects of enhanced intestinal ME1 expression, in the context of HF diet, on serum levels of insulin, leptin, adiponectin, and blood glucose levels, all of which constitute markers of insulin sensitivity/resistance and glucose homeostasis. At 12 wk of age (9 wk on HF diet), ME1-Tg mice displayed a significant increase in fasting blood glucose levels (Figure 2C), however, this difference was not maintained at 18 wk of age. However, two-way ANOVA of blood glucose levels for the combined weeks 12 and 18 revealed a significant effect of genotype (*P* = 0.041), but not of time-points. Serum levels of insulin, leptin, and adiponectin did not differ between WT and ME1-Tg mice of 18 wk of age, although there was a tendency *(P* = 0.07) for increased insulin resistance as reflected by the HOMA-IR index (Figure 2D–F).

### Intestinal ME1 enhances intestinal epithelial cell proliferation during diet-induced obesity

**Figure 3:**
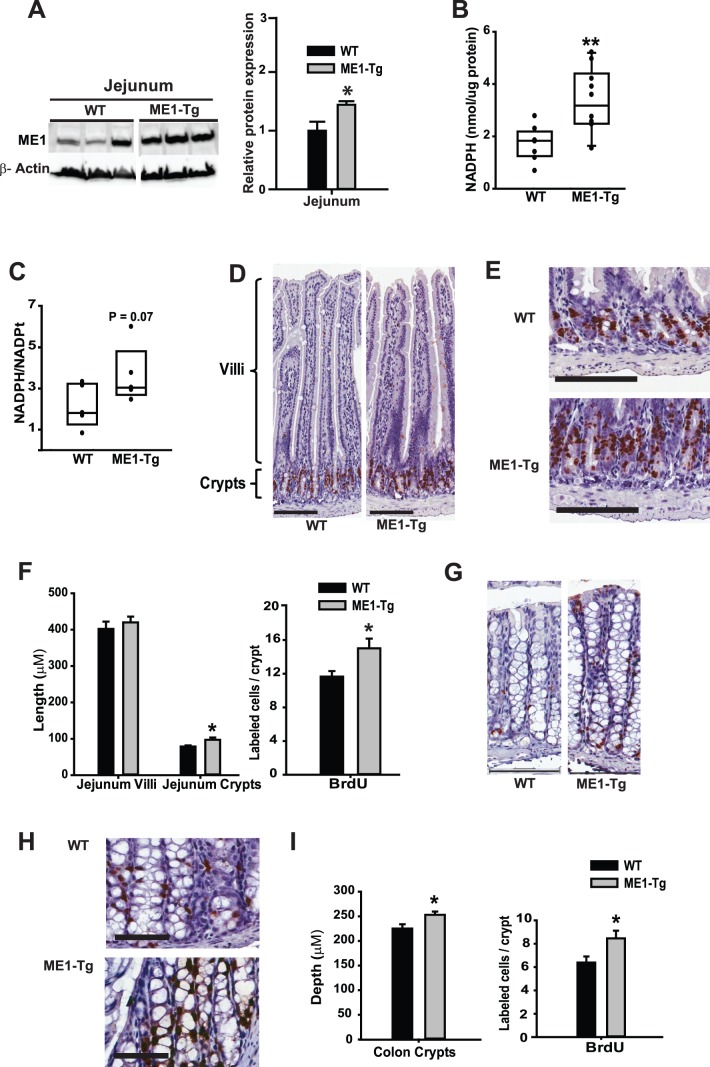
Increased crypt cell proliferation in jejunums of ME1-Tg mice fed HF diet. A) Western blot of ME1 protein and corresponding band densitometry analysis of WT and ME1-Tg mice fed HF diet (Exp. 2). B) ME1 enzyme activity in the jejunum of WT and ME1-Tg mice. Each data point represents an individual mouse. C) Tissue ratio of NADPH/NADPt in jejunum samples of WT and ME1-Tg mice. D–E) Representative images of jejunum villi, crypts, and BrdU staining of WT and ME1-Tg mice; scale bars = 100 µM (D and E). F) Quantification of jejunum villus height and crypt depth, and BrdU staining of jejunum of WT and Me1-Tg mice (Exp. 2). G–H) Representative images of colon crypts, and BrdU labeling of WT and ME1-Tg mice; scale bars = 100 µM (G) and 50 µM (H). I) Quantification of colon crypt depth, and BrdU staining of colons of WT and Me1-Tg mice (Exp. 2). Significant differences at *P<0.05 and ** *P*<0.01 between genotypes. *P* values are indicated for those values tending to exhibit significant differences between groups (0.05<*P*<0.10). For panels B, C, F and I, data were from 5–6 mice/group.

To determine the effect of enhanced ME1 expression on intestinal epithelial cell proliferation, we evaluated small intestine morphology and BrdU labeling of crypt cells in the HF diet-fed WT and Tg mice (Exp. 2). For this objective, we focused on the jejunum since it is the major intestinal site of lipid processing and absorption, and its phenotype is markedly affected by HF diet [2]. Western blots showed a ∼1.5-fold elevation of ME1 in jejunum of ME1-Tg mice (Figure 3A), which paralleled increased jejunal ME1 enzyme activity and greater tissue NADPH/NADPt content (Figure 3B–C). ME1-Tg mice displayed increased jejunum crypt depth and greater numbers of BrdU-labeled crypt cells compared to WT counterparts (Figure 3D–F), albeit no differences in villus height were observed. ME1-Tg mice similarly displayed increased colon crypt depth and greater numbers of BrdU-labeled crypt cells compared to WT counterparts (Figure 3G–I). Results demonstrate promotion of intestinal crypt cell proliferation by ME1.

### Intestinal expression of lipogenic, cholesterologenic, and proliferation-associated genes is augmented in ME1-Tg mice

**Figure 4:**
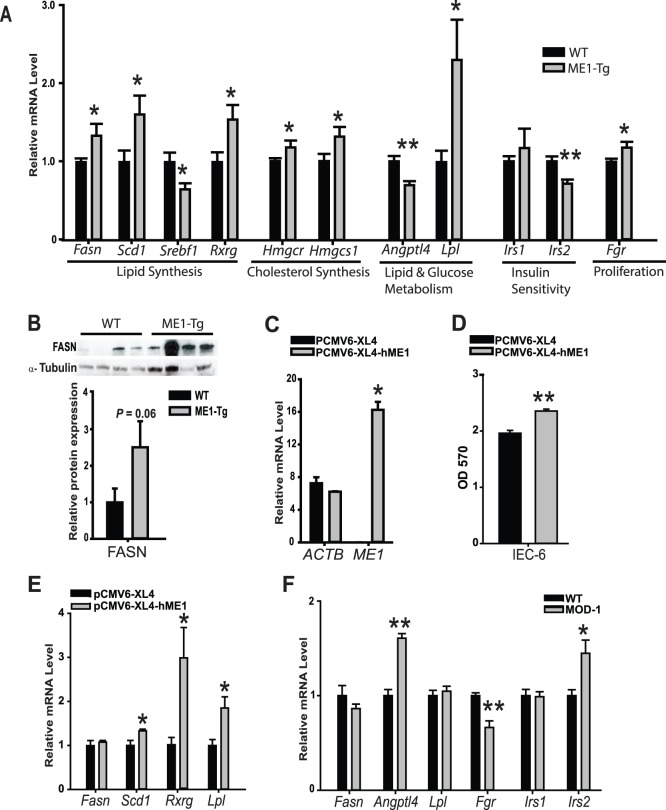
Enhanced intestinal ME1 expression with HF diet induces jejunum lipogenic- and proliferation-associated gene expression. A) Relative expression of jejunum genes of WT and ME1-Tg mice [Exp. 2 (n = 7−9/group)]. B) Western blot and corresponding band densitometry analysis of FASN in the jejunum of WT and ME1-Tg mice (Exp. 2). C–E) Effects of ME1 over-expression on intestinal epithelial cell proliferation and gene expression *in vitro*. Rat intestinal epithelial cells (IEC-6) were transfected with control or hME1 expression vectors; overexpression of human ME1 mRNA, but not beta-actin mRNA, was observed by RT-PCR (C). At 48 h, cells were evaluated for proliferation by MTT assay (D), and at 96 h (E) evaluated for expression of *Fasn*, *Scd1*, *Rxrg* and *Lpl* genes (n = 3 replicates/group). F) Gene expression of *Fasn*, *Angptl4*, *Lpl*, *Fgr*, *Irs1* and *Irs2* in jejunums of WT and MOD-1 mice fed HF diet (n = 5/group). RT-PCR was repeated twice in all experiments, and MTT proliferation assay was repeated thrice. Bar graphs represent mean ± SEM. Significant differences at *P<0.05 and ** *P*<0.01 between genotypes.

We next probed for effects of enhanced ME1 activity on expression of several lipogenic, cholesterologenic, and proliferation-related genes. Jejunums of ME1-Tg *vs.* WT mice were evaluated for differential gene expression by qRT-PCR (Figure 4A). Transcript levels of Fatty Acid Synthase (*Fasn*), Stearoyl CoA Desaturase 1 (*Scd1*), Retinoid X Receptor Gamma (*Rxrg*), Lipoprotein lipase (*Lpl*), HMG-CoA Reductase (*Hmgcr*), 3-hydroxy-3-methylglutaryl-CoA Synthase 1 (*Hmgcs1*), and Gardner-Rasheed Feline Sarcoma viral (*Fgr*) oncogene homolog were all up-regulated in jejunums of ME1-Tg mice. By contrast, mRNA abundance of Angiopoetin-like Factor 4 (*Angptl4*), a negative regulator of LPL activity, was significantly reduced in ME1-Tg relative to WT littermates. In addition, Sterol Regulatory Element-Binding Protein 1c (*Srebf1*) mRNA expression was significantly reduced in the jejunum of ME1-Tg *vs.* WT mice. The induction of *Fasn* mRNA in ME1-Tg mice was confirmed at the level of the corresponding protein (Figure 4B). On the other hand, Western blotting and immunohistochemical staining of LPL in jejunum did not mimic the difference in mRNA levels noted between WT and ME1-Tg mice (Figure S3A–C), consistent with LPL being a secreted protein. Insulin receptor substrate (*Irs1, Irs2*) mRNA levels also were evaluated in WT and Tg mice jejunums. While *Irs2* gene expression was reduced in ME1-Tg mice, consistent with an attenuated state of insulin sensitivity (Figure 4A), *Irs1* mRNA abundance did not differ between genotypes.

To confirm the direct effects of enhanced ME1 expression on epithelial cell proliferation and gene expression noted *in vivo*, we transfected rat intestinal epithelial IEC-6 cells with an expression vector for human ME1 or corresponding control vector, and measured cell proliferation/cell viability by MTT assay. Human ME1 mRNA over-expression was confirmed by qRT-PCR of transfected cells (Figure 4C). ME1-transfected cells exhibited increased proliferation compared to the control vector-transfected cells (Figure 4D). Moreover, ME1-transfected cells showed higher *Scd1*, *Rxrg* and *Lpl* transcript levels, while that for *Fasn* did not change (Figure 4E) in ME1-transfected, compared to control cells.

We previously reported that mice functionally null for ME1 (MOD-1 mouse line) are protected from diet-induced obesity and exhibit reduced cell proliferation in colon and small intestine [15]. We therefore determined whether absence of ME1 affected jejunum gene expression in a manner opposite to that found for ME1-Tg mice, when both were compared to corresponding WT counterparts. Indeed, expression of *Angptl4* and *Irs2* genes was up regulated, while that of *Fgr* was reduced in MOD-1 mice (Figure 4F); changes that were in the opposite direction from those observed for ME1-Tg mice (Figure 4A). However, expression of *Fasn* and *Lpl* mRNAs in the jejunums of MOD-1 mice did not differ from those of corresponding WT mice (Figure 4F).

### Intestinal ME1 promotes expression of hepatic genes associated with lipogenesis, cholesterol synthesis and cholesterol uptake

**Figure 5:**
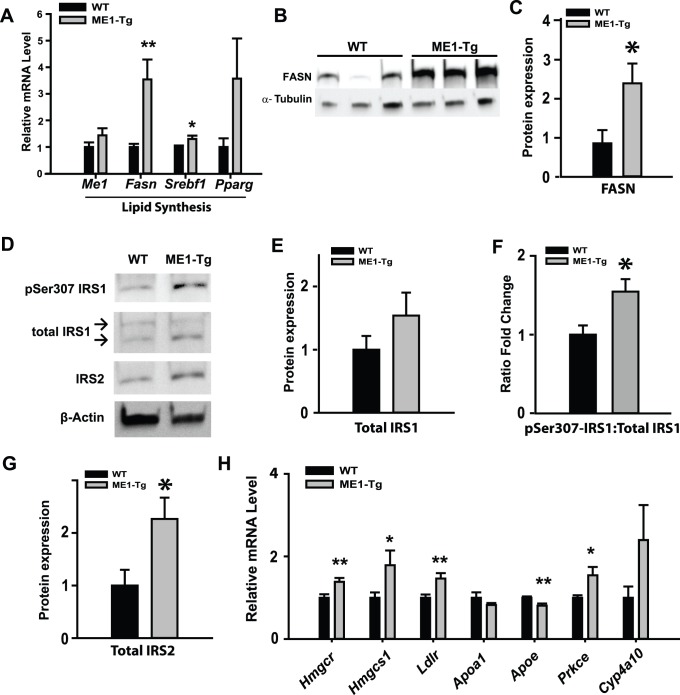
Expression of lipogenic pathway and cholesterol synthesis pathway genes in livers of WT and ME1-Tg mice fed HF diet. A) qRT-PCR analysis of major lipogenic pathway genes in livers of WT and ME1-Tg mice fed HF diet (Exp. 2; n = 8−9/group). B) Western blot of FASN protein in livers of WT and ME1-Tg mice fed HF diet. C) Densitometric analysis of immunoreactive bands in (B) relative to α-Tubulin protein. D) Western blot of IRS1, pSer307-IRS1, and IRS2 in livers of WT and ME1-Tg mice fed HF diet (n = 5/group). E–G) Densitometric analysis of immunoreactive bands of total liver IRS1 (E) and IRS2 (G) and the relative ratio of immunoreactive pSer307-IRS1/total IRS1 band densities (F). H) mRNA expression of select cholesterol synthesis- and cholesterol uptake-related genes in the livers of WT and ME1-Tg mice (Exp. 2; n = 8−9/group). qRT-PCR reactions were repeated twice in all experiments (A, H). Bar graphs represent mean ± SEM. Significant differences at *P<0.05 and ** *P*<0.01 between genotypes.

The greater liver weights of ME1-Tg mice (Exp. 2) suggested possible changes in liver metabolic phenotype. To evaluate this possibility, we examined the relative expression of a number of lipogenic genes in livers of WT and ME1-Tg mice (Exp. 2). qRT-PCR analysis showed significant increases in the mRNA abundance of *Fasn, Srebf1*, *Hmgcr*, *Hmgcs1*, *Prkce* and *Ldlr* (Ldl Receptor), decreased *Apoe* expression, and unchanged expression of *Apoa1 and Cyp4a10* in livers of ME1-Tg compared to WT controls (Figure 5A, H). These changes in liver gene expression in ME1-Tg mice occurred without a parallel change in endogenous liver *Me1* expression (Figure 5A) and in the absence (as expected) of *Me1* transgene expression in liver (Figure S4). Western blot analysis confirmed the increase in FASN levels in ME1-Tg liver (Figure 5B–C). Moreover, while total hepatic IRS1 levels were not significantly altered as a function of genotype, ME1-Tg mice exhibited a greater ratio of pSer307-IRS1 to total IRS1 than for WT controls (Figure 5D–F). An increase in hepatic IRS2 expression was similarly observed in ME1-Tg relative to WT mice (Figure 5G). Results suggest that intestinal ME1 levels indirectly mediate liver gene expression, metabolism and insulin sensitivity.

### Intestinal ME1 and susceptibility to hepatosteatosis

**Figure 6:**
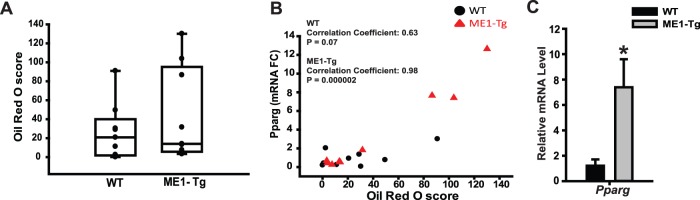
Liver lipid content correlates with *Pparg* gene expression in mice fed HF diet. A) Quantification of Oil Red O staining intensity of liver samples using Aperio Image Scope software. B) Pearson’s correlation analysis for the level of liver mRNA expression of *Pparg* with Oil Red O score of WT and ME1-Tg mice. C) Comparisons of liver mRNA levels of *Pparg* in WT and ME1-Tg mice that exhibited Oil Red O score of >20; n = 4−5/group. Bar graphs represent mean ± SEM. Significant differences at *P<0.05 between genotypes. *P* value is indicated for that tending to have a significant difference between groups (0.05<*P*<0.10).

In animal models of diet-induced obesity, increased hepatic expression of lipogenic and cholesterol metabolism-related genes and proteins normally precedes the development of hepatosteatosis and non-alcoholic fatty liver disease (NAFLD) [25], [26]. We therefore evaluated lipid accumulation (by Oil Red O staining) in livers of WT and ME1-Tg mice (Exp. 2). For these studies, liver sections from mice of each genotype were analyzed. In the ME1-Tg group, 3 of 9 mouse livers displayed severe and diffuse macro- and micro-vesicular steatosis in areas surrounding the portal triad and hepatic vein, inflammatory foci, and intense Oil Red O staining. By contrast, only 1 of 9 WT mouse livers conformed to these descriptions (Figure 6A and Figure S5). The degree of steatosis was positively correlated with mRNA expression of *Pparg* in livers of both genotypes; however, ME1-Tg mice displayed a stronger correlation (Figure 6B). Statistically significant differences in hepatic *Pparg* mRNA levels between Tg and WT mice were not evident, however analysis of the data only for mice that showed moderate to severe steatosis (Oil Red O score >20) in both groups showed a significant increase in *Pparg* in Tg mice compared to WT mice (Figure 6C). Serum cholesterol and triglyceride levels and liver cholesterol content for all animals, however, did not significantly differ with genotype (Figure S6A–B).

## Discussion

Obesity and associated co-morbidities involve multiple organ systems and are affected by dietary and genetic factors [27]–[29]. Intestinal epithelial cells, which play an important role in digestion, absorption and transport of nutrients, are responsive to biochemical mediators that influence energy storage and metabolism [7], [30]. Nevertheless, their role in obesity has been under-evaluated since their primary function is not lipogenesis. Recent studies provide indications that molecular aberrations in small intestine phenotype may influence the development of overweight/obesity and associated pathologies. For example, elevated expression of ME1 and other lipogenic genes in the small intestine of HF diet-induced obese animal models parallels phenotypic changes that occur in their liver and adipose tissues [2], [4], [5]. ME1, a major lipogenic enzyme, is considered to play an important role in obesity and associated pathologies [15]–[17], but its ubiquitous tissue expression precludes complete understanding of whether and how it may regulate metabolic parameters in intestine and liver as well as lipid handling between these tissues. We began to address these questions by generating a new transgenic mouse line with enhanced ME1 expression in small intestine villous epithelium. Using this new mouse model, we identified phenotypic and functional changes in intestines, which were accompanied, by gene expression changes in liver.

Transgenic mice expressing rat ME1 in the intestinal epithelium under the control of the murine villin1 promoter-enhancer (ME1-Tg mice), and with higher than normal overall intestinal ME1 activity, manifested an increase in body weight but only when fed HF diet. In the context of HF diet, Tg mouse weight gain was exacerbated, due in part to increased liver but not gonadal or retroperitoneal fat depot, weights. The intestinal ME1-directed liver phenotype was accompanied by increased hepatic IRS1 activation in Tg mice, consistent with the development of whole body insulin resistance [31] and with increased mRNA expression of Protein Kinase C-ε, known to be associated with hepatic insulin resistance [32]. Further, small intestines and livers of Tg mice exhibited elevated expression of a subset of key lipogenic and cholesterologenic genes, indicative of coordinate alterations in tissue metabolic status. Collective results provide support for intestinal ME1 impacting liver lipid and cholesterol metabolism and the regulatory linkage between these two tissues to influence obesity and insulin resistance.

Small intestine epithelial cells exhibit a high turnover rate, and a remarkable capacity to adapt to nutritional status [33]–[35]. These cells display reduced proliferation during the fasted state, and conversely, increased proliferation in the fed state, with dietary lipids generally serving as proliferative stimuli. Our findings that jejunums and colons from Me1-Tg mice showed increased crypt BrdU incorporation and that a rat intestinal epithelial cell line overexpressing ME1 displayed elevated proliferation/cell viability are consistent with our previous report that mice null for ME1 (MOD-1 mouse line) exhibited reduced expression of lipogenic and proliferation-associated genes (*Fasn*, Cyclin D1 and *Ki67*) in the colon and small intestine compared to control mice [15]. The mechanisms underlying the proliferative role of ME1 in intestinal epithelial cells have not been extensively explored, but may involve pro-proliferative and/or anti-apoptotic mechanisms [36], [37]. FGR, a member of the Src family of tyrosine kinases, functions as a regulator of cell migration and adhesion. This protein is overexpressed in many solid tumors, lymphomas and leukemias; plays an important role in tumor growth; and is associated with aggressive tumor features [38],[39]. In the jejunum, ME1-Tg mice exhibited increased *Fgr* expression, while MOD1 mice exhibited reduced *Fgr* expression, compared to WT counterparts. These intriguing observations are consistent with the observed increase in jejunum crypt cell proliferation in the ME1-Tg mice and conversely, reduced intestinal proliferation in the MOD-1 mice. Little is known about the function of FGR in intestinal epithelium; however, our data implicate ME1 in proliferation of these cells and suggest an intriguing possible link with *Fgr*. Future studies should examine these apparent linkages in both fed and fasted states and as a consequence of obesogenic diet. A recent paper reported a reciprocal regulation of malic enzymes (ME1 and ME2) with p53 in the modulation of metabolism and senescence in normal fibroblast cells and in cancer cells, wherein ME1 overexpression delayed senescence and accelerated growth [21]. It is also possible that the known HF diet-elicited induction of intestinal ME1 expression (9; 10; 29) may drive stem-progenitor cell proliferation in response to fat consumption; although this remains to be tested.

Jejunum, a major tissue site of fatty acid and cholesterol processing, absorption and synthesis [40]–[42], manifested a set of genes that were differentially expressed in ME1-Tg *vs.* WT mice and which may underlie the ME1-Tg phenotype. Transcripts for lipogenic proteins were up-regulated in the jejunum of ME1-Tg mice, likely leading to an increased state of *de novo* fatty acid synthesis with enhanced ME1 expression. FASN is necessary for intestinal epithelial proliferation and barrier maintenance, and is up-regulated in liver and adipose tissue of obese subjects [43]. Stearoyl-CoA desaturase-1 (SCD1) catalyzes the conversion of saturated long-chain fatty acids into monounsaturated fatty acids (MUFA), the latter being major components of triglycerides, cholesterol esters and phospholipids, and having important roles in lipid metabolism [44], [45]. Increased expression of SCD1 in the muscles of obese patients and rats leads to insulin resistance, while its deficiency in mice improves insulin sensitivity and prevents hepatosteatosis [46]. RXR Gamma (RXRG) is a nuclear transcription factor that heterodimerizes with other nuclear receptors such as liver X receptors (LXRs) and peroxisome proliferator-activated receptors (PPARs) to mediate gene transcription [47], [48]. RXRG is known to be involved in lipid and glucose metabolism [49]. Over-expression of RXRG in skeletal muscle increased triglyceride content [50]. Thus, the novel associations, both *in vivo* and *in vitro*, of intestinal ME1 with intestinal *Fasn*, *Scd1*, and *Rxrg* warrant follow-up studies to examine the nature of these associations (direct or indirect), and possible involvement of malate, pyruvate, and NADPH/NADP^+^ ratio.

Fat storage in adipose and liver tissue involves many transporters and enzymes as well as cross-talk between tissues via the circulation, and can involve intestinal host-microbe interactions that modify the response to nutrients of key genes involved in lipid metabolism [8], [30], [51]. ANGPTL4 is a ubiquitously expressed and secreted protein involved in lipid metabolism and energy storage, through its inhibition of lipoprotein lipase (LPL) and pancreatic lipase enzyme activity and stimulation of lipolytic gene expression. This protein is inversely associated with obesity, hyperlipidemia, hyperglycemia and insulin resistance in humans and rodent models [52]–[55]. LPL is a multifunctional enzyme produced by many tissues that facilitates the hydrolysis of triglycerides from circulating lipoproteins and chylomicrons to promote their uptake and storage by these tissues [56]. Our findings that ME1-Tg mouse jejunum manifested reduced expression of *Angptl4* and increased *Lpl* expression, whereas MOD-1 mice had higher jejunal *Angptl4* expression compared to WT counterparts, support the involvement of intestinal ME1 in regulating intestinal and systemic lipid and glucose metabolism in part through ANGPTL4.

Based on the above observations, we examined the expression of *Irs1* and *Irs2* genes, since metabolic alterations usually precede the development of tissue insulin resistance [57]–[60]. ME1-Tg mice displayed reduced levels of *Irs2* mRNA in the jejunum indicative of a reduced state of insulin sensitivity [61]; conversely, MOD-1 mice exhibited increased jejunum *Irs2* mRNA levels indicating a higher state of insulin sensitivity. While the observed increase in HOMA-IR index in ME1-Tg mice paralleled the reduction in *Irs2* expression in the jejunum, levels of pSer307 IRS1 were increased in the liver of ME1-Tg mice, consistent with increased insulin resistance secondary to hepatic lipid and cholesterol accumulation in the liver. This alteration in insulin sensitivity may have been partially compensated for by increased levels of liver IRS2, protecting from further liver lipid accumulation and steatosis. Notably, the enhanced liver IRS1 activation concomitant with increased blood glucose in ME1-Tg mice suggests the onset/development of systemic insulin resistance [62]. Consistent with our present findings, a compensatory increase in IRS2 expression was previously demonstrated to accompany loss of hepatic IRS1, leading to increased fatty acid metabolism [31], [63]. Such alterations in liver IRS1 and IRS2 may account for observed increases in blood glucose in the absence of increased liver fatty acid levels. The transient nature of elevations in blood glucose may be attributed to continuous high-fat diet consumption. It is possible that intestinal ME1 overexpression affects blood glucose in the initial stages of diet-induced obesity, but that this effect is not maintained because chronic high-fat diet may eventually cause frank hyperglycemia, thus, obviating the genotype effect. We suggest that ME1 mediates tissue insulin resistance, in part, by affecting tissue steady-state levels of IRS proteins.

**Figure 7:**
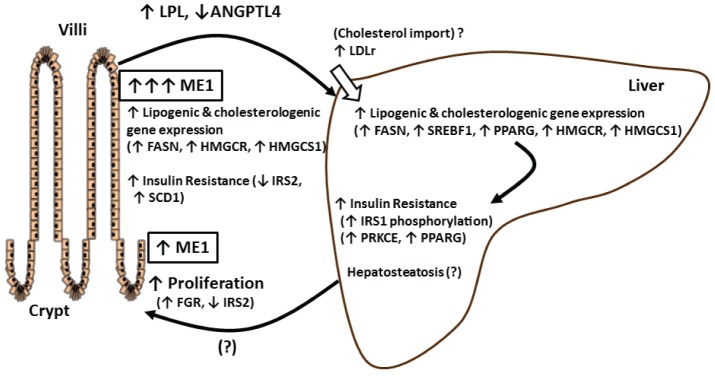
Proposed model summarizing the major findings and implications of this study. (?) Represents current unknowns.

Our proposed summary model (Figure 7) integrates altered intestinal metabolism due to enhanced intestinal ME1 with elevated lipogenic and cholesterol synthesis gene expression in the liver, which may increase susceptibility to hepatosteatosis, resulting in multi-organ insulin resistance [57], [58]. Excess hepatic fat accumulation (hepatosteatosis) is a hallmark of non-alcoholic fatty liver disease (NAFLD) [64], [65]. Greater expression of lipogenic *Fasn* and *Srebf1* and cholesterologenic *Hmgcr* and *Hmgccs1* genes point to, but do not prove, increased *de novo* lipogenic and cholesterologenic states in the liver consequent to augmented intestinal ME1 expression. Interestingly, this seemed to only be the case in the context of HF diet consumption, as these changes were not exhibited in chow diet-fed animals. Nevertheless, the enhanced expression of *Pparg* gene and its greater correlation with the degree of steatosis and pathological changes in ME1-Tg mice are likely indicative of deregulated cholesterol metabolism and increased reactive oxygen species in the liver [66] and consistent with up-regulated *Pparg* expression in the steatotic livers of obese patients and mouse models [67], [68]. Since the liver steatosis phenotype was not fully penetrant in WT and ME1-Tg mice, a longer duration of HF diet feeding and larger number of animals in each group may be required to test the likelihood of increased risk/propensity for NAFLD in the Tg mice.

In summary, enhanced expression of ME1 in the gastrointestinal epithelium resulted in increased intestinal crypt cell proliferation and altered expression of fatty acid- and cholesterol- biosynthetic pathway genes. Such changes were accompanied by alterations in hepatic expression of lipogenic and cholesterologenic genes as well as a shift in markers of insulin sensitivity. The nature of the molecular signal(s) by which the small intestine influences liver metabolic programs consequent to enhanced intestinal ME1 expression awaits identification. Our results highlight a new mouse model that should prove useful in addressing the role of small intestine ME1 in whole body metabolism, hepatomegaly and hepatosteatosis, and crypt cell proliferation.

## Supporting Information

Figure S1 **ME1-Tg mice on chow diet do not exhibit change in liver or fat pad weights compared to WT.** Weights of A) livers, B) both gonadal fat pads, and C) both retroperitoneal fat pads of WT and ME1-Tg male mice (n = 8−10 mice/group) at 11 wks. D) Fasting blood glucose levels of WT and ME1-Tg male mice (n = 8−10 mice/group) at 11 wks. Circulating serum E) insulin and F) leptin of WT and ME1-Tg male mice (n = 7 mice/group) at 11 wks. Bar graphs represent mean ± SEM. Box plots represent median values with upper and lower quartiles.
(EPS)

Figure S2 **mRNA expression of select lipogenic (A) and cholesterol synthesis/cholesterol uptake-related and other (B) genes in the livers of WT and ME1-Tg mice (Exp. 1; n = 8−9/group).** Bar graphs represent mean ± SEM. Significant differences at *P<0.05 between genotypes.
(EPS)

Figure S3 **ME1-Tg mice on HF diet do not exhibit change in jejunum expression of lipoprotein lipase (LPL) compared to WT when fed HFD.** A) Representative Western blot of LPL protein in the jejunum. (n = 4/group; Exp. 1). B) Densitometric analysis of relative ME1 protein levels in panel A. C) Representative images of LPL immunostaining in WT and ME1-Tg mouse jejunums. Scale bars = 50 µM.
(EPS)

Figure S4 **mRNA expression of the ME1 transgene in the jejunums and livers of WT and ME1-Tg fed HF diet mice as measured by RT-PCR.**
(EPS)

Figure S5 **Representative images from Oil Red O-stained WT and ME1-Tg livers after HF diet.** Each image represents a different mouse liver. Scale bars = 100 µM.
(EPS)

Figure S6 **Cholesterol and triglyceride levels did not differ between genotype when fed HF diet.** A) Serum cholesterol levels (n = 9 mice/group) and B) liver cholesterol levels (n = 5−6 mice/group). Box plots represent median values with upper and lower quartiles.
(EPS)
